# Contributing to health security in Europe since 2005 – ECDC’s 15th anniversary

**DOI:** 10.2807/1560-7917.ES.2020.25.20.2000975

**Published:** 2020-05-21

**Authors:** Andrea Ammon

**Affiliations:** 1 Director, European Centre for Disease Prevention and Control (ECDC), Stockholm, Sweden

**Keywords:** public health, infectious diseases, Europe

Coronaviruses appear to mark milestones for the European Centre for Disease Prevention and Control (ECDC). It was the sudden emergence of the severe acute respiratory syndrome coronavirus (SARS-CoV) in 2003 that led to the establishment of ECDC which took up its work on 20 May 2005 in Solna, Sweden [1]. Today, on its 15-year anniversary, a new coronavirus, SARS-CoV-2 and the associated coronavirus disease (COVID-19), is dominating the day-to-day work of the Centre and of health services around the globe.

The mission and the build-up of ECDC around its core functions and disease-specific work were described in detail on the occasion of its 10th anniversary in 2015. Since then, the Centre has grown into a mature organisation and it became firmly established as a reliable and trusted partner of the European Union (EU) Institutions, the EU and European Economic Area (EEA) countries and internationally operating organisations in the field of communicable disease prevention and control, such as the World Health Organization (WHO). In pursuing its legal mandate to identify, assess and communicate threats to human health from emerging and current infectious diseases [2], several topics emerged as priorities for ECDC in the past 5 years.

Immunisation is a highly successful intervention to prevent communicable diseases with an evident need to sustain or achieve high vaccination coverage. However, decreasing coverage in some countries for important childhood vaccinations such as against measles, is threatening elimination plans as evidenced by outbreaks and very high numbers of cases in the years 2017 to 2019, in Europe [3]. A worrying example of the negative influence of mistrust in the safety and benefits of immunisation has been that of the controversies around vaccination against human papilloma virus (HPV) that led to a sharp decline of HPV vaccination coverage in some countries [4]. The reasons for the delay or refusal of vaccines are complex and vary depending on the context and vaccines. They include factors such as complacency, convenience and confidence. In order to support restoring the confidence and based on earlier work, ECDC published a catalogue of interventions addressing vaccine hesitancy [5] and developed toolkits to strengthen the skills of healthcare workers in their dialogue with parents having doubts about vaccinating their children [6]. Acknowledging the growing need to re-establish trust in immunisation where confidence has been lost, the Council of the European Union issued a recommendation on strengthened cooperation against vaccine-preventable diseases in December 2018 [7]. ECDC supports the implementation of this recommendation with expert advice and various activities. Examples are the recently launched multilingual European Vaccination Information Portal with evidence-based information on questions around vaccination, and the collaboration of European National Immunisation Technical Advisory Groups (NITAGs) established in 2018. This collaboration aims to share and generate scientific evidence on the use of EU-authorised vaccines and immunisation practices, with ECDC providing the secretariat. For effectiveness and safety studies conducted to inform NITAGs and immunisation programmes, the expanded use of electronic immunisation registries supported by ECDC to document/validate vaccinations has been a welcome development [8,9].

Antimicrobials are the other important tools to curb the morbidity and mortality from infectious diseases. The silent epidemic of antimicrobial resistance, in particular the increasing resistance to last-line antibiotics, globally threatens achievements made in the past and requires interdisciplinary joint action in a ‘One Health’ approach to preserve the effectiveness of antimicrobial treatments. A pertinent example of such collaboration is that of the Joint Interagency Antimicrobial Consumption and Resistance Analysis (JIACRA) reports by ECDC, European Medicines Agency (EMA) and European Food Safety Authority (EFSA) resulting from the European Commission Action Plan against the rising threats from antimicrobial resistance (AMR) [10,11]. Infections acquired in the healthcare setting mainly affect vulnerable population groups such as the elderly, those with underlying diseases, children and frail individuals. Antimicrobial stewardship and adequate infection prevention and hygiene measures are important tools to lower the burden of this important public health threat. As healthcare workers are key in implementing these measures, ECDC published the results of a survey of healthcare workers’ knowledge, attitudes and behaviours on antibiotics with more than 18,000 participants from 30 EU/EEA countries [12]. This first study of its kind, lays the foundations for evidence-based actions. In line with its mission, ECDC applied and further developed important methodologies for estimating the burden of AMR and healthcare-associated infections at European and national levels [13,14]. These data are key in identifying specific areas of action and informing policy making.

Outbreaks of vector-borne diseases outside of Europe such as chikungunya, dengue and Zika virus have repetitively led to mostly smaller outbreaks in Europe often with the source case being a returning viraemic traveller. West Nile virus has become established in several southern and some central European countries with increasing geographical expansion. Preparedness and integrated approaches combining human and animal surveillance as well as vector control activities and collaborations with diverse partners are essential in responding to the threat of new vector-borne diseases becoming established within Europe. ECDC has established a network of laboratories, the Emerging Viral Diseases-Expert Laboratory Network (EVD-LabNet), a European network of expert laboratories supporting ECDC in early detection and surveillance of (re)emerging viral diseases in the EU/EEA, and in providing scientific advice [15]. A joint initiative of EFSA and ECDC, VectorNet, supports the collection of data on vectors and pathogens in vectors, related to both animal and human health, implementing the ‘One Health’ perspective for this work [16].

The thus far largest Ebola virus disease epidemic in West Africa starting in 2013 and ending in 2016, required a major international public health response. ECDC mobilised 62 experts from EU countries, the European Programme for Intervention Epidemiology Training (EPIET) and the European Programme for Public Health Microbiology, and from ECDC, who were deployed through the WHO Global Outbreak Alert and Response Network mechanism. This marked the first large scale international deployment of ECDC experts. Shortcomings identified in the response led to the establishment of the European Medical Corps that includes public health teams, a component that ECDC is coordinating [17]. ECDC experts were also involved in the recent outbreak in the Democratic Republic of the Congo, where a newly developed vaccine applied predominantly through ring vaccinations, was a major factor for controlling the outbreak in a region struck by a long-lasting armed conflict.

International collaboration is key to increased health security through exchange of experience and information as well as support to countries to enhance their capacities and capabilities to detect, assess, respond, and prevent health threats caused by infectious diseases. Aside from a close collaboration with partners in the European Commission and within the EU/EEA, ECDC has established international cooperation with national public health institutes in the EU Enlargement and European Neighbourhood Policy (ENP) partner countries, with national and regional Centers for Disease Control (CDC) in North America, Asia and the newly established African CDC. As part of the accession process, ECDC conducted at the request of the European Commission, assessments of EU Enlargement countries’ infectious disease prevention and control systems, supported countries in the development of action plans, and organised regional workshops on e.g. performance of public health laboratories and antimicrobial resistance. Nominated experts from these countries are invited to ECDC meetings to successively acquaint them with ECDC’ networks. ECDC, through funding from the European Commission, is working with the ENP partner countries to strengthen health security through capacity building e.g. via the Mediterranean Programme for Intervention Epidemiology Training and activities related to preparedness.

As one major development that has rapidly transformed the way epidemiologists perform investigations and analyses, whole genome sequencing (WGS) has become routine in the majority of countries in Europe. An important advantage of WGS is a higher resolution in outbreak investigations that allows identifying links in situations where few cases manifest in geographically dispersed areas. In particular, cross-border outbreak identification is facilitated by comparison of sequences, preferably through public accessible databases. ECDC has progressively facilitated sequencing for food-borne and other pathogens since 2015 and developed a strategic framework for integrating WGS into EU surveillance and multi-country outbreak investigations. A respective roadmap is now successively implemented.

None of ECDC’s activities would be possible without collaboration in our networks of EU/EEA experts, in particular with colleagues from the national public health institutes. The continuous dialogue and exchange on needs and expected support from ECDC, borne by a mutual dedication to public health and willingness to continuously refine methods and advance technological approaches, gives ECDC’s work relevance and focus.

The ongoing COVID-19 pandemic is having a profound impact on public health and healthcare systems worldwide. Evaluations and after-action reviews to translate the experience of this pandemic into strengthening the preparedness for such events in the future are still to come. Although the outcomes are not yet on the table, observations from the first 4 months of the pandemic allow a few conclusions. The massive consequences on the global movement of people and goods and the economies of all countries remind us that rather than just being a cost, public health and preparedness to crises constitute one of the most important investments our societies should ensure.

We need robust surveillance systems at EU and national level that provide reliable and timely data also in a crisis situation. An increased level of digitalisation will make parts of the surveillance continuum independent from the time public health experts can spend on it. Experiences currently gathered, for example with contact tracing Apps, can feed into future effective and feasible solutions. Other elements would include further implementation of electronic health records that contain parameters important for public health, the application of artificial intelligence for data validation and analysis, and automated reporting. Despite available preparedness plans in all EU/EEA countries, hospital preparedness will require reviewing and strengthening, in terms of monitoring beds, human capacities, stockpiles of essential medicines and equipment. As consequence of possible changes, the skillset of public health workers will need to be adapted and new training priorities will emerge.

International coordination and cooperation have been reaffirmed as critical aspects of handling and controlling the pandemic. This is relevant for countries in the neighbourhood of the EU and the rest of world. Rapid sharing of information, data and experience is key to avoid that each country or region has to go through the same learning process. The potential detrimental effect of the necessary focus of the health systems on COVID-19, on other diseases, infectious and non-infectious, is not yet visible. Part of strengthening preparedness plans may mean to clearly establish essential parts of national health programmes that need to be preserved as long as possible, even during a crisis.

ECDC’s establishment was sparked by the SARS outbreak, and nearly two decades later, ECDC’s future will certainly be influenced by the lessons learned from the COVID-19 pandemic. ECDC was conceived as ‘small, but influential’. ECDC has increased its influence and visibility to the extent possible with the current mandate and resources, especially during the COVID-19 pandemic; a thorough evaluation of the way ECDC acted during the pandemic should give recommendations as input for discussions on adaptations of ECDC’s role and size in the future.
